# Gamified behavioral engagement as an indirect association linking ideal L2 self and vocabulary achievement in mobile-assisted EFL vocabulary learning

**DOI:** 10.3389/fpsyg.2026.1836665

**Published:** 2026-05-28

**Authors:** Jie Pan, Jie Liang, Yanan Hu

**Affiliations:** Shanghai Normal University Tianhua College, Shanghai, China

**Keywords:** EFL learners, gamified behavioral engagement, ideal L2 self, indirect pathway, mobile-assisted vocabulary learning

## Abstract

**Introduction:**

Gamified mobile-assisted language learning (MALL) has increasingly been used to support vocabulary learning by encouraging learner participation and sustained practice. However, evidence regarding the effectiveness of gamification remains mixed, and the processes through which motivational resources are associated with vocabulary learning outcomes remain insufficiently investigated. In particular, limited research has examined how motivational self-guides and behavioral engagement may jointly relate to vocabulary achievement in mobile-assisted learning contexts. Drawing on the L2 Motivational Self System (L2MSS) and engagement research in second language acquisition, the present study examined a statistical indirect pathway linking the Ideal L2 Self (IL2S), gamified behavioral engagement (GBE), and vocabulary achievement.

**Methods:**

A two-wave, classroom-based observational study was conducted with first-year Chinese EFL students (*N* = 181) over a 13-week semester. Structural equation modeling (SEM) was employed while controlling for baseline vocabulary knowledge.

**Results:**

IL2S significantly predicted GBE (*β* = 0.359, *p* < 0.001), and GBE was positively associated with vocabulary achievement (*β* = 0.120, *p* = 0.007). By contrast, the direct relationship between IL2S and vocabulary achievement was not significant (*β* = 0.052, *p* = 0.185). Bootstrapping analyses revealed a statistically significant indirect association through behavioral engagement (*β* = 0.043, 95% CI [0.012, 0.096]).

**Discussion:**

These findings suggest that, in a gamified mobile-assisted vocabulary learning context, IL2S was indirectly associated with vocabulary performance through sustained behavioral engagement, whereas its direct association with achievement was not statistically significant.

## 1 Introduction

Vocabulary learning is widely recognized as a fundamental component of second language development (Nation, 2022; Schmitt, 2008). Meanwhile, vocabulary development can be demanding for L2 learners because it involves acquiring large numbers of lexical items as well as multiple aspects of word knowledge, often through repeated exposure, review, and use over time (Nation, 2022; Schmitt, 2008). Sustaining such effort may be challenging, particularly when vocabulary practice is experienced as routine or repetitive. Against this background, gamification has increasingly been incorporated into digital learning environments as a design approach intended to make learning activities more engaging and to encourage continued participation (Deterding et al., 2011; Koivisto and Hamari, 2019). Gamification generally refers to the use of game design elements, such as points, leaderboards, and progress indicators, in non-game contexts (Deterding et al., 2011). In foreign language education, these elements are commonly embedded into instructional tasks in order to support learner participation and persistence (Koivisto and Hamari, 2019). Empirical studies have similarly suggested that gamified tools and mobile-assisted language learning applications can help learners sustain vocabulary practice and remain involved in learning beyond the classroom (Fithriani, 2021; Waluyo and Bucol, 2021; Rojabi et al., 2022; Jarrah et al., 2025).

However, the effectiveness of gamified learning environments should not be overstated. Although meta-analytic evidence suggests that gamification is generally associated with positive learning outcomes, its effects are not uniformly strong, and the impact on behavioral learning outcomes in particular is often modest (Sailer and Homner, 2020). A similar caution is reflected in Dehghanzadeh et al. (2019) systematic review of digital gamification for learning English as a second language. The review found that gamified environments were frequently associated with enjoyable, engaging, and motivating learning experiences, as well as with outcomes such as content language learning, engagement, motivation, and satisfaction. However, it also noted that the literature had not clearly specified which gamification elements were linked to which outcomes. This inconsistency has led scholars to question not only whether gamification works, but also how it works. As Alsawaier (2018) noted, gamification research has often reported positive motivational or engagement-related outcomes without clearly specifying how these learner responses are associated with learning outcomes. In a similar vein, Koivisto and Hamari (2019) argued for more process-oriented research that clarifies how psychological and behavioral factors interact in technology-enhanced learning environments. In the context of language learning, this suggests a need to move beyond broad claims about the benefits of gamification and to examine more specifically how motivation may be associated with achievement through learners’ behavioral engagement in learning activities.

One useful framework for addressing this issue is the L2 Motivational Self System (L2MSS; Dörnyei, 2005), which conceptualizes language learning motivation in relation to learners’ future self-guides. Within this framework, the Ideal L2 Self (IL2S) refers to a learner’s desired future image as a competent L2 user and is regarded as an important motivational resource for sustaining long-term language learning effort (Boo et al., 2015; Dörnyei, 2005). Previous research has shown that learners with a stronger IL2S tend to display higher levels of engagement across language learning activities (Gong and Pang, 2025). At the same time, however, motivational self-guides operate at a relatively distal level. Their relevance to achievement is therefore likely to depend on more immediate forms of learner involvement, especially sustained behavioral engagement in actual learning tasks (Oga-Baldwin et al., 2019; Zhan et al., 2023). This distinction between distal motivational resources and more proximal engagement processes is particularly relevant in gamified learning environments, where learners’ continued participation may be as important as their initial motivational orientation.

This issue may be especially salient in mobile-assisted vocabulary learning (MAVL). Vocabulary development depends heavily on repeated exposure, retrieval, and cumulative practice over time, which makes learners’ sustained behavioral involvement particularly important (Nation, 2022; Lin and Lin, 2019). Although gamified applications may provide a context that encourages repeated participation, relatively little research has examined how learners’ future-oriented motivational self-guides are associated with vocabulary achievement through behavioral engagement in such environments. To address this gap, the present study examines whether gamified behavioral engagement (GBE) is statistically involved in an indirect association linking IL2S and vocabulary achievement in a gamified MAVL context.

## 2 Literature review

### 2.1 Gamification: definition and applications in foreign language education

In technology-enhanced language learning, gamification is commonly discussed not simply as the addition of isolated game elements, but as a pedagogical design logic through which specific affordances may shape learners’ psychological responses and sustained behavioral engagement. Points, badges, leaderboards, levels, rewards, and immediate feedback can provide learners with visible goals, progress cues, and reinforcement during repeated learning activities (Deterding et al., 2011; Wulantari et al., 2023). From this perspective, gamified learning is expected to support vocabulary learning not because game elements are inherently effective, but because they may make repetitive practice more structured, goal-directed, and behaviorally sustainable. This distinction is particularly relevant in mobile-assisted vocabulary learning, where learners’ continued participation across multiple learning sessions may be more consequential than short-term interest alone. Whether these affordances are empirically associated with measurable vocabulary-learning outcomes, however, remains an empirical question that requires further examination.

In foreign language education, gamification has been used particularly in areas that involve repeated practice, such as vocabulary learning. Recent review-based work suggests that gamified instruction is generally associated with positive learner responses in EFL/ESL contexts, particularly in terms of motivation, participation, and perceived learning support (Chan and Lo, 2024). In classroom-based settings, tools such as Kahoot have been used to promote active participation, concentration, and enjoyment during language tasks, with evidence from EFL vocabulary instruction indicating gains in engagement, motivation, and quiz performance (Rojabi et al., 2022). Related findings from other subject domains likewise suggest that Kahoot-based instruction may support students’ motivation and performance, although such evidence should not be treated as foreign-language-specific support (Jarrah et al., 2025). In mobile-assisted language learning, gamified vocabulary applications have also been reported to support vocabulary development while being perceived by learners as enjoyable and motivating (Fithriani, 2021). Related evidence from app-based vocabulary learning likewise suggests that gamified conditions may be associated with higher learning outcomes, motivation, and learner satisfaction than non-gamified alternatives in some contexts (Yu, 2023). Similarly, gamified vocabulary instruction incorporating teamwork, competition, rewards, and leaderboards has been found to strengthen learners’ motivation, although learners do not respond equally positively to all such features (Sadeghi et al., 2022). Evidence from L2 vocabulary learning also suggests that gamified instruction can sustain learner engagement while yielding lexical gains that are at least comparable to those achieved through more traditional classroom activities (Cancino Avila and Castillo Fonseca, 2022).

At the same time, the literature does not support treating gamification as a uniformly effective instructional approach. Review-based work has repeatedly noted that its effects vary across contexts, target outcomes, and instructional designs (Alsawaier, 2018; Chan and Lo, 2024). Meta-analytic evidence further indicates that gamification tends to have positive effects on learning outcomes overall, but the magnitude of these effects differs by outcome type, with behavioral outcomes often smaller than cognitive or motivational ones (Sailer and Homner, 2020). This variability has led researchers to move beyond asking whether gamification is simply effective and instead to examine which affordances work, for whom, and under what conditions (Koivisto and Hamari, 2019). A similar point emerges from Dehghanzadeh et al. (2019) systematic review of gamified digital LESL research, which concluded that although positive learning experiences and outcomes were frequently reported, the available studies did not clearly specify which particular gamification elements were linked to those experiences and outcomes. One reason is that learners do not respond identically to the same mechanics. For example, research on gamification user types suggests that features such as competition, social discovery, or team-based interaction may be experienced differently depending on learner preferences and motivational profiles (Bovermann and Bastiaens, 2020). In a similar vein, more recent design-oriented work emphasizes that gamified systems should be aligned with learning objectives, activity types, and learner characteristics rather than implemented as one-size-fits-all solutions (Palomino et al., 2023).

In summary, the current literature suggests that gamification in foreign language education is best understood not as a universally effective instructional formula, but as a contextual design approach whose value depends on how specific mechanics are embedded into pedagogical practice. In this aspect, relatively simple mechanics—such as task quantification, progress visualization, points, and immediate feedback—may be especially relevant in MAVL because they can support repeated practice without requiring elaborate game worlds or complex narrative structures (Deterding et al., 2011; Fithriani, 2021; Koivisto and Hamari, 2019). Consistent with this perspective, the present study focuses on these foundational gamified affordances in a naturally occurring mobile-assisted learning environment and examines how they are related to learners’ self-reported behavioral engagement, rather than assuming that all forms of gamification function in the same way.

### 2.2 Ideal L2 self as a motivational resource for vocabulary learning

Within the L2MSS, the IL2S refers to learners’ future-oriented self-image of themselves as competent users of the target language (Dörnyei, 2005). Since the introduction of the L2MSS, this construct has occupied a prominent position in L2 motivation research. A large-scale review of publications from 2005 to 2014 showed that the period following the proposal of the L2MSS was marked by a substantial expansion of research on L2 motivation, with self-related constructs becoming especially visible in the field (Boo et al., 2015). More broadly, this development also reflects a shift in motivation research from earlier macro-social perspectives toward greater attention to contextual, dynamic, and long-term aspects of language learning, including the role of technology in everyday learning environments (Al-Hoorie, 2017).

This perspective is relevant to mobile-assisted vocabulary learning, where progress often depends on whether learners can sustain repeated engagement over time. Some recent studies suggest that digital or game-related learning environments may be associated with more positive self-perceptions in language learning. For example, Liu et al. (2024) found that learners in a digital gamified language learning condition outperformed those in a non-digital condition not only in language achievement and enjoyment but also in IL2S. Likewise, Zhou (2024), in a Duolingo-based digital game-based learning context, reported that digital game-based learning significantly enhanced learners’ enjoyment and IL2S-perception while also examining how these variables evolved during the learning process. Taken together, these studies indicate that technology-enhanced and gamified learning conditions may be compatible with more positive future-oriented L2 self-perceptions in some contexts, although the available evidence does not justify a stronger claim that such environments uniformly strengthen the IL2S across settings (Liu et al., 2024; Zhou, 2024).

From a theoretical standpoint, the IL2S is often treated as a productive motivational resource because it is rooted more in personal aspiration than in external obligation. By contrast, the ought-to L2 self is defined more explicitly in relation to external expectations and the avoidance of undesirable outcomes (Dörnyei, 2005). Recent evidence from L2 writing research is broadly consistent with this distinction: Zhu et al. (2026) found that the IL2S positively predicted self-competence and writing performance, whereas the ought-to L2 self was more closely linked to externally grounded forms of self-evaluation and showed no significant direct effect on writing performance. This suggests that the IL2S may be more conducive to adaptive, self-endorsed motivation, whereas the motivational consequences of the ought-to dimension appear more complex and less consistently facilitating (Zhu et al., 2026).

At the same time, a future self-image alone is unlikely to explain learning outcomes unless it is related to learners’ actual involvement in learning activity. In language education, engagement has been described as a central construct connecting learners’ prior beliefs, abilities, and experiences with subsequent learning outcomes (Oga-Baldwin et al., 2019). Empirical evidence from L2 writing research also points in this direction. Gong and Pang (2025) found that the ideal L2 writing self was positively associated with multiple dimensions of task engagement and significantly mediated the relationship between transactional writing beliefs and behavioural, cognitive, emotional, social, and agentic engagement. Although this evidence comes from writing rather than vocabulary learning, it supports the broader proposition that a desired future L2 self may matter partly because it is associated with learners’ active engagement in learning tasks.

On this basis, it is reasonable to view the IL2S in gamified mobile-assisted vocabulary learning as an important motivational resource rather than as a direct guarantee of achievement. In such contexts, learners who can imagine themselves as more capable future L2 users may be more likely to remain involved in repeated vocabulary practice, although the strength and form of this association are likely to depend on the learning context and the way engagement is measured. Accordingly, in the present study, the IL2S is conceptualized as a motivational antecedent that may be associated with vocabulary development through its relationship with learners’ self-reported behavioral engagement in gamified learning activities.

### 2.3 IL2S and self-reported behavioral engagement

Student engagement is commonly conceptualized as a multidimensional construct comprising behavioral, emotional, and cognitive dimensions (Fredricks et al., 2004). In language learning research, this multidimensionality has been further elaborated to reflect the situated and task-dependent nature of learners’ involvement, with different dimensions often interacting rather than operating in isolation (Philp and Duchesne, 2016). This tripartite view has also been adopted in recent technology-enhanced EFL research. For example, Shen et al. (2024) examined student engagement in an SVVR-supported online EFL writing class and showed that behavioral, emotional, and cognitive engagement could be enhanced both independently and interdependently in a technology-mediated language-learning environment. Recent L2 engagement research has likewise emphasized that engagement is dynamic and context-sensitive rather than a fixed learner trait (Zhou et al., 2023). At the same time, these dimensions are not interchangeable, and they need not play identical roles in explaining learning processes or outcomes.

The present study focuses specifically on behavioral engagement. In prior literature, behavioral engagement has generally been associated with learners’ effort, attention, persistence, and active participation in learning activities (Fredricks et al., 2004; Skinner and Belmont, 1993; Furrer and Skinner, 2003). In L2 engagement research, it is likewise treated as the tangible behavioral investment that learners bring to classroom or task participation. For example, Zhou et al. (2023) describe engagement as requiring concrete student attention and participation beyond initial desire or motivation, and further relate stronger engagement to higher levels of attention, focus, persistence, and effort. In this sense, behavioral engagement may be understood as the more observable or self-reported action component of learner involvement, whereas cognitive and emotional engagement refer more to mental processing and affective reactions, respectively (Philp and Duchesne, 2016; Gong and Pang, 2025). This narrower focus is appropriate in the present context because mobile-assisted vocabulary learning depends heavily on whether learners continue returning to repeated practice over time. In such settings, behavioral engagement can therefore be treated as a relatively direct indicator of sustained involvement in learning activity, even though it does not exhaust the broader construct of engagement.

From the perspective of the L2MSS, the IL2S is typically understood as a future-oriented motivational resource rather than as a behavior in itself. Its relevance to achievement is therefore expected to depend, at least in part, on whether it is associated with more immediate forms of learner involvement. Existing studies in L2 writing provide support for this general logic, although the specific behavioral pathways under examination differ across studies. For example, Zhan et al. (2023) conceptualized feedback-seeking behavior as a self-initiated learning behavior and showed that ideal L2 writing self positively predicted such behavior, with the indirect path to performance differing across proficiency groups. Similarly, Gong and Pang (2025) found that ideal L2 writing self was positively associated with multiple dimensions of task engagement, including behavioral engagement, although its strongest association in that study was not behavioral but emotional. These findings support a cautious claim that future self-guides may matter partly because they are associated with learners’ active involvement in learning tasks.

This does not mean, however, that behavioral engagement should be treated as wholly separate from affective experience. Philp and Duchesne (2016) explicitly note the interdependence among dimensions of engagement and discuss emotions as activating or deactivating engagement. Likewise, Oga-Baldwin et al. (2019) describe engagement as a central construct linking learners’ prior beliefs, abilities, and experiences with later outcomes. Zhou et al. (2023) similarly treat engagement as part of a dynamic classroom process rather than as an isolated behavioral residue. Taken together, these works suggest that behavioral engagement should not be conceptualized in isolation from emotion or cognition. Rather, in contexts requiring repeated self-directed practice, learners’ effort, persistence, attention, and continued participation provide a meaningful indicator of how a future-oriented motivational self-guide may be associated with ongoing learning activity.

At the same time, the distinction between the ideal and ought-to dimensions should be stated cautiously. Zhu et al. (2026) found that the IL2S positively predicted self-competence and writing performance, whereas the ought-to L2 self showed a more complex and less consistently facilitative pattern. Although that study did not directly examine behavioral engagement, it still supports the more limited view that the IL2S may function as a more adaptive motivational resource than the ought-to L2 self in some L2 learning contexts. Accordingly, the present study conceptualizes IL2S not as a direct behavioral variable, but as a motivational antecedent that may be associated with vocabulary development through its relationship with learners’ self-reported behavioral engagement in gamified learning activities.

### 2.4 Self-reported gamified behavioral engagement in MAVL

MAVL is particularly relevant to vocabulary development because vocabulary learning is cumulative and typically requires repeated encounters with lexical items over time. Mobile applications are useful not simply because they are digital, but because they can extend vocabulary learning beyond limited class time into out-of-class settings. Recent synthesis work also suggests that mobile applications can support vocabulary learning effectively, especially in longer interventions, although outcomes are not uniformly positive across all studies or app types (Hao et al., 2021; He and Loewen, 2022; Xu and Richardson, 2024; Zhou and Zhou, 2026).

In the present study, self-reported gamified behavioral engagement (GBE) is not treated as mere time spent on an app; rather, it refers more specifically to learners’ reported effort, persistence, on-task attention, and continued participation in gamified vocabulary-learning activities. This framing aligns with prior work treating behavioral engagement as the action-oriented component of motivation, reflected in learners’ willingness to initiate and sustain learning activity (Furrer and Skinner, 2003; Skinner and Belmont, 1993). GBE is conceptualized as a context-specific form of behavioral engagement within mobile app-based vocabulary learning rather than as a broad indicator of general classroom engagement. Related evidence from technology-enhanced EFL learning lends further support to this behavioral interpretation: in an SVVR-supported online EFL writing class, Shen et al. (2024) found that technology-mediated tasks could foster learners’ careful participation, active interaction with learning materials, and on-task attention, which closely reflect the behavioral dimension of engagement as conceptualized here.

Self-reported GBE was used as the primary measure because it captures learners’ perceived effort, attention, and persistence, aspects not fully reflected in fine-grained app logs such as time on task, number of completed words, review frequency, item-level accuracy, or response latency. For example, time on task may include passive or distracted use, whereas high completion frequency may partly reflect compliance with course requirements. Accordingly, self-reported GBE and behavioral logs should be regarded as complementary rather than interchangeable indicators.

This decision is consistent with how behavioral engagement has been operationalized in app-based vocabulary learning research. Prior studies have employed self-report scales, log-based indicators, and task-participation records, collectively suggesting that behavioral engagement can be meaningfully examined through different operationalizations depending on instructional and technological contexts. He and Loewen (2022), for example, operationalized engagement through actual app use, highlighting the persistent challenge of sustaining engagement in app-based L2 learning. Similarly, Li et al. (2025) used a context-specific scale to capture learners’ attention and behavioral involvement in AVL tasks, supporting self-reported engagement as a meaningful construct. Sensitivity analyses using weekly check-in score confirmed that self-reported GBE captures meaningful variance in learner engagement beyond compliance-based app use.

A gamified MAVL environment may provide such support through specific affordances, but claims must remain measured. For example, Al-Hoorie and Albijadi (2024) found that badges, points, instant feedback, and goal-oriented rewards provided a posttest advantage for the gamified condition, though not at delayed posttest, suggesting gamification may enhance short-term behavioral involvement but does not guarantee uniform or durable engagement. Xu and Richardson (2024) reported that badge-sharing promoted self- and shared regulation, emphasizing that vocabulary learning is incremental, largely outside class, and requires sustained time investment. Thus, self-reported GBE reflects learners’ ongoing willingness to return to app-based vocabulary tasks, maintain attention, and persist with repeated practice, rather than serving as a simple proxy for achievement.

The relationship between gamification and such behavioral involvement is also likely to be conditional rather than uniform. Recent work has emphasized that the same gamification strategies may produce different outcomes for different learners and that one-size-fits-all designs can be less effective than more personalized approaches (Bovermann and Bastiaens, 2020; Palomino et al., 2023). Bovermann and Bastiaens (2020) explicitly connect different gamification mechanics with different user types, while Palomino et al. (2023) argue that applying the same strategy to all learners may yield uneven effects and that poorly designed personalization may even hinder learning. For this reason, the present study does not assume that gamification automatically produces engagement; rather, it examines whether learners with a stronger IL2S report higher levels of behavioral engagement within a gamified vocabulary-learning environment, and whether such engagement is in turn associated with vocabulary achievement.

### 2.5 Self-reported behavioral engagement and vocabulary achievement

From a learning perspective, self-reported behavioral engagement may be expected to show a positive association with vocabulary achievement, especially in MAVL contexts where improvement depends on repeated practice over time. Recent evidence from Chinese university EFL learners suggests that classroom engagement is positively associated with English academic achievement (Tang et al., 2026), lending broader support to the view that engagement may represent a relevant proximal correlate of achievement in EFL learning. Prior work on app-based L2 vocabulary learning suggests that this relationship is plausible, but not always straightforward. He and Loewen (2022), for example, note that vocabulary self-study through apps can support vocabulary development, yet the link between app-based vocabulary activity and broader proficiency outcomes appears less straightforward. In their study, instructional support increased the number of words studied, but did not produce significant differences in TOEIC scores. This suggests that behavioral engagement may matter, but its effects are likely to be clearer when the outcome measure is closely aligned with the practiced vocabulary behaviors rather than when it reflects broader and more distal proficiency. More specifically, Cancino Avila and Castillo Fonseca (2022) reported that a gamified vocabulary-learning approach increased learners’ engagement while producing immediate and delayed vocabulary gains that were at least as high as those observed under traditional classroom instruction. Although this study did not model behavioral engagement as a mediator, it supports the more limited proposition that engagement-related advantages in gamified vocabulary contexts may coexist with meaningful lexical development.

A similarly cautious interpretation is warranted in the broader MAVL literature. Zhou and Zhou (2026) report a generally positive overall effect of mobile applications on vocabulary learning and delayed retention, suggesting that sustained use of mobile tools can be beneficial for vocabulary development. At the same time, their meta-analysis also identifies variation across moderators, indicating that the strength of this effect is not uniform across all conditions. These findings support the view that sustained behavioral engagement can be beneficial for vocabulary learning, but they do not justify a simple one-to-one assumption that more engagement will always translate into stronger achievement across contexts.

Evidence from gamified vocabulary studies also points to a generally positive but still qualified engagement–achievement relationship. Rojabi et al. (2022) found that Kahoot-supported vocabulary instruction was associated with higher vocabulary exam scores as well as stronger engagement and motivation. Students also described the quizzes as helping them participate, concentrate, and review course content more actively. However, the authors also acknowledge contextual constraints such as small sample size, internet problems, and task difficulty. Thus, the study supports a positive connection between engagement and performance, but not an unqualified generalization.

By contrast, Sadeghi et al. (2022) provide a useful reminder that higher motivation and more positive perceptions in a gamified vocabulary condition do not necessarily translate into significantly greater vocabulary gains. In their study, the gamified group reported motivating features such as competition, fun, and teamwork, but the gain scores between the gamified and control groups did not differ significantly. This makes it more accurate to say that engagement-favorable conditions may support vocabulary learning, while the size and detectability of achievement differences depend on how the learning activity is designed and how achievement is assessed.

For this reason, it is more rigorous to treat behavioral engagement as a proximal indicator of learner involvement rather than as a direct synonym for achievement. Review-based work on gamification has repeatedly emphasized that outcomes vary according to context, learner characteristics, and the design of the affordances themselves. Koivisto and Hamari (2019), for instance, argue that gamification research should move beyond simply asking whether gamification works and pay closer attention to the contextual and individual factors that shape its effects on motivation and behavior. This perspective fits the present study well: self-reported behavioral engagement is best understood as one construct that may be associated with vocabulary performance, rather than as a uniform effect of gamification itself.

Hence, in the present study, the association between self-reported behavioral engagement and vocabulary achievement is interpreted as a theoretically consistent relationship rather than a strong causal claim. The available literature supports the expectation that learners who report greater persistence, effort, and continued involvement in vocabulary practice may also show better vocabulary outcomes, particularly when learning activities and assessment are closely aligned. At the same time, prior findings also make clear that this relationship can be attenuated by short intervention periods, broader outcome measures, or contextual constraints in the learning environment.

### 2.6 Vocabulary pre-test as a covariate

Vocabulary pre-test score was incorporated into the structural model as a covariate to account for learners’ baseline differences in prior vocabulary knowledge. Because vocabulary learning is cumulative, post-test performance may reflect not only learning during the intervention but also learners’ initial level of vocabulary knowledge. In educational modeling, prior achievement is commonly included as a covariate when predicting later achievement, allowing researchers to obtain baseline-adjusted estimates of subsequent performance (Levy et al., 2019). This adjustment is particularly useful in naturally occurring instructional contexts, where learners may differ in their initial proficiency before the intervention begins.

In addition, vocabulary pre-test score was specified as covarying with IL2S. In the present model, this covariance was included to account for shared variance between learners’ initial vocabulary knowledge and their motivational self-perceptions, rather than to imply a directional causal relationship. This specification is also consistent with previous research suggesting that learners’ actual language proficiency is related to the formation and plausibility of their ideal L2 self-images (Wong, 2020). By incorporating vocabulary pre-test score as a covariate and allowing it to covary with IL2S, the model estimates associations among motivational self-guides, behavioral engagement, and vocabulary achievement while accounting for baseline differences in prior vocabulary knowledge.

### 2.7 Summary and research hypotheses

According to the reviewed literature, the IL2S remains a prominent construct in L2 motivation research and continues to be relevant to contemporary, technology-mediated learning environments (Boo et al., 2015; Al-Hoorie, 2017). At the same time, engagement is widely understood as a multidimensional construct, within which behavioral engagement refers more specifically to learners’ effort, persistence, and participation and can therefore be distinguished conceptually from emotional and cognitive engagement (Fredricks et al., 2004; Philp and Duchesne, 2016). In MAVL, vocabulary development depends heavily on sustained practice over time, although the strength of the engagement–achievement relationship may vary according to outcome measures, intervention duration, and other contextual factors (He and Loewen, 2022; Zhou and Zhou, 2026).

Within this framework, IL2S may be understood as a future-oriented motivational resource rather than as a behavior in itself. Prior work has highlighted the central role of engagement in connecting learners’ prior beliefs and experiences with subsequent learning outcomes (Oga-Baldwin et al., 2019), while recent EFL research has further shown that engagement may serve as a proximal pathway through which contextual resources are associated with achievement (Tang et al., 2026). Related L2 research also suggests that stronger future self-images are associated with higher levels of learner engagement across tasks and contexts (Gong and Pang, 2025). At the same time, research in mobile vocabulary learning has not, to our knowledge, sufficiently integrated IL2S, self-reported GBE, and vocabulary achievement within a single explanatory model. To address this gap, the present study examines whether self-reported GBE is associated with the relationship between IL2S and performance-type vocabulary achievement in a naturally occurring gamified MAVL context.

*H*1: IL2S is positively associated with self-reported GBE.

*H*2: Self-reported GBE is positively associated with vocabulary achievement.

*H*3: IL2S is positively associated with vocabulary achievement.

*H*4: IL2S shows a statistical indirect association with vocabulary achievement via self-reported GBE.

## 3 Methodology

### 3.1 Research design and participants

This study employed a two-phase quantitative design. The first phase focused on the adaptation and psychometric validation of the measurement instruments in a MAVL context, whereas the second phase involved a two-wave, classroom-based observational study conducted over a 13-week semester to examine a baseline-adjusted statistical indirect-association model linking IL2S, GBE, and vocabulary achievement.

Phase 1 data were collected in late June 2025. A total of 245 non-English-major undergraduate students were recruited from multiple universities in the Yangtze River Delta region of China using snowball sampling. All participants were regular users of mobile applications for English vocabulary learning, ensuring that the validation context closely matched the intended instructional setting. Participants were aged between 18 and 22 years, including 104 males (42.45%) and 141 females (57.55%). This sample was used exclusively for the adaptation and validation of the Chinese version of the IL2S scale and the contextualized behavioral engagement scale, and it did not overlap with the participants in the field study. All Phase 1 participants were regular users of MAVL applications, ensuring that the scale validation context was ecologically compatible with the gamified MAVL environment examined in the subsequent field study.

Phase 2 was conducted during the fall semester of 2025 at a private university in eastern China using convenience sampling. The sample consisted of 181 first-year non-English-major students from six intact teaching-administrative classes, aged between 18 and 20 years, including 48 males (26.52%) and 133 females (73.48%). All participants were enrolled in the compulsory course *College English I*. The study was reviewed and approved by the Institutional Review Board (IRB) of the university. All participants were informed of the purpose and procedure of the study before data collection, and informed consent was obtained from all students prior to participation.

### 3.2 Measurement tools

Learners’ IL2S was measured using a Chinese-adapted version of the L2MSS questionnaire originally developed by Taguchi et al. (2009). The original items were translated and culturally adapted to ensure clarity, linguistic accuracy, and contextual relevance for Chinese non-English-major university students engaged in MAVL. Responses were recorded on a six-point Likert scale ranging from 1 (*strongly disagree*) to 6 (*strongly agree*). Psychometric evaluation based on the Phase 1 sample indicated excellent internal consistency, with a Cronbach’s alpha coefficient of 0.932 and item-level reliability coefficients exceeding 0.90. Confirmatory factor analysis further supported the construct validity of the adapted IL2S scale, yielding satisfactory model fit indices (χ^2^/df = 1.94, CFI = 0.991, TLI = 0.982, RMSEA = 0.062, SRMR = 0.022).

Students’ self-reported GBE was measured using a four-item adapted scale developed for the present gamified mobile-assisted vocabulary learning context, informed by Zhou et al. (2023) and subsequent context-specific research. Item wording was revised to capture learners’ attention, effort, persistence, and continued participation in app-based vocabulary learning activities. Participants responded on a seven-point Likert scale ranging from 1 (*completely inconsistent with me*) to 7 (*completely consistent with me*). Four items were used to assess students’ behavioral engagement in this process. An example item is “I pay attention when learning vocabulary with the app.” Validation based on the Phase 1 sample demonstrated high internal consistency (Cronbach’s *α* = 0.936), with all items showing reliability coefficients above 0.90. Confirmatory factor analysis results also indicated satisfactory construct validity (χ^2^/df = 2.17, CFI = 0.984, TLI = 0.979, RMSEA = 0.069, SRMR = 0.037). Together, these results indicate that both adapted instruments were psychometrically sound and suitable for subsequent SEM-based indirect-association analysis. Consistent with the construct definition, GBE was conceptualized as learners’ perceived behavioral investment, rather than a simple count of app-use events. Self-reported GBE and weekly check-in records were treated as complementary indicators: check-in scores documented participation but did not capture the quality of attention, effort, or persistence. For this reason, self-reported GBE served as the primary measure, with weekly check-in used as supplementary evidence and examined in sensitivity analyses.

Vocabulary achievement was assessed using two researcher-developed CET-4-based vocabulary tests aligned with the CET-4 Syllabus Vocabulary List (abridged version) embedded in the Shanbay vocabulary learning app, reflecting the vocabulary-learning content practiced during the semester. The two forms were designed as comparable curriculum-based measures rather than standardized parallel tests. Item selection considered the CET-4 list, the vocabulary-learning content assigned during the intervention, and the instructional level of first-year non-English-major students, representing a range of semantic difficulty levels and word classes consistent with CET-4 vocabulary instruction.

The initial item pool and final test forms were reviewed by two experienced EFL instructors with doctoral degrees and extensive College English teaching experience. They evaluated whether the selected target words, sentence contexts, distractors, and answer keys were appropriate for first-year non-English-major students, and whether the two forms were broadly comparable in syllabus coverage, item format, content relevance, and expected difficulty. Minor revisions were made based on their feedback, supporting content validity and instructional appropriateness, although no separate pilot testing was conducted.

Both tests followed the same two-part structure. Part I included 25 multiple-choice items assessing receptive word meaning, and Part II included 25 word-bank cloze items assessing contextualized vocabulary use. Each item was worth two points, yielding a total possible score of 100. Answer keys were fixed, and responses were scored as correct or incorrect with no partial credit. The pre-test contained 50 target items with no repeats. The post-test also had 50 items, 10 of which were repeated target words assessed through different items, contexts, and response options; the remaining 40 were newly selected from the same CET-4 list. This partial-overlap design maintained content continuity while minimizing direct practice effects. The pre-test score served as a baseline covariate, and the post-test score was the vocabulary achievement outcome in the mediation model.

Since repeated words were tested in new contexts, the two forms are not fully independent parallel tests, but they share specifications, content domain, and scoring structure. Observed differences in mean scores reflect both learning gains and form-difficulty variation, justifying use of the pre-test as a covariate rather than a simple difference score.

### 3.3 Procedure

In Phase 2, data collection followed a two-wave design over one academic semester lasting 13 weeks. At Time 1 (the first class session of the semester), participants completed the adapted IL2S questionnaire and the vocabulary pre-test. From late September 2025 onward, all six classes were required to use the Shanbay application outside regular class time to study the same CET-4 Syllabus Vocabulary List (abridged version, 2,060 target words) as a required out-of-class component of the compulsory College English I course. All students used the same vocabulary list, followed the same weekly learning requirement, and received the same general instructions.

After selecting the assigned CET-4 vocabulary list in Shanbay, students set a fixed weekly learning plan in the app. They were required to study 160 newly assigned words per week, with each session consisting of 40 newly assigned words and 80 review words (1:2 ratio). “Newly assigned words” refers to words first presented within the Shanbay learning plan rather than words necessarily unknown to students before the intervention. Students could choose any 4 days within each week to complete the required sessions, with four learning days representing the minimum course requirement. Since the assigned list contained 2,060 words, the final week was adjusted so that students completed the remaining words rather than exactly 160 newly assigned words. This arrangement provided scheduling flexibility while maintaining a consistent minimum learning load across the 13-week intervention.

At the beginning of the intervention, course instructors explained the purpose of the Shanbay-based vocabulary task, the required weekly learning frequency, the app-setting procedure, the weekly check-in documentation procedure, and the role of the task in continuous assessment. All classes received the same instructions, vocabulary list, learning load, app settings, and monitoring procedure.

In terms of its learning design, Shanbay was treated as a gamified mobile-assisted vocabulary learning environment because it embedded selected game design elements into routine vocabulary practice. These features did not turn vocabulary learning into a full-fledged game; rather, they introduced goal-setting, task quantification, progress visualization, feedback, and achievement-oriented reinforcement into a non-game vocabulary-learning context. Table 1 summarizes the specific gamified affordances of Shanbay relevant to the present study, along with their operational manifestations and intended engagement-related functions.

**Table 1:** Gamified affordances of Shanbay used in the present study.

Gamified affordance	Operational manifestation in Shanbay	Engagement-related function
Daily check-in	After completing the assigned vocabulary-learning task, students could use the app-based check-in function to record task completion. The check-in function made daily completion visible to learners and provided a record of documented participation.	Encouraged routine participation, continuity, and sustained task completion.
Progress tracking	The app displayed students’ learning progress and task completion status, including the number of words learned, overall progress within the selected vocabulary book, and indicators of word mastery.	Supported goal monitoring, progress awareness, and self-regulation during vocabulary learning.
Immediate feedback	During vocabulary learning and review, the app provided immediate feedback based mainly on learners’ recognition responses, such as whether they reported knowing or not knowing a word and whether the response matched the target meaning.	Supported response adjustment, repeated review, and attention to difficult or insufficiently mastered words.
Achievement- or badge-like rewards	The app included achievement-oriented indicators or badge-like rewards for continued participation, such as badges for consecutive check-ins or vocabulary-learning milestones.	Reinforced persistence, continuity, and a sense of accomplishment in repeated vocabulary learning.
Task quantification	Weekly vocabulary learning goals were operationalized into concrete daily or weekly targets, such as the number of new words and review words to be completed.	Made learning requirements concrete, monitorable, and easier to translate into regular learning behavior.

Shanbay was conceptualized as a mobile-assisted vocabulary learning app incorporating selected gamified affordances rather than as a fully game-based learning environment. The affordances listed here refer to app-embedded features that structured, monitored, and reinforced students’ vocabulary learning behaviors during the intervention.

To provide a practical behavioral record of students’ app use, a class-level weekly check-in monitoring procedure was adopted. Around the beginning of each week, class monitors collected check-in evidence from students regarding their Shanbay learning in the previous week, recorded documented completion status, and submitted class-level summaries to the course instructor. For assessment purposes, each valid daily check-in record was awarded 2 points and a missing record 0 points; since the minimum requirement was four learning days per week, students could receive up to 8 check-in points per week. Check-in completion accounted for 15% of the overall course grade to encourage regular participation across the semester.

These check-in records served as a practical but coarse indicator of documented participation. They reflected recorded task completion but did not capture fine-grained learning behavior such as time on task, cumulative word completion, review frequency, item-level accuracy, or response latency. Since Shanbay is a commercial application operated by a third-party provider, the research team had no administrative access to the platform’s backend, and individual-level behavioral logs were proprietary and unavailable under the platform’s terms of use. In addition, because check-in completion was tied to course assessment, the records may have partly reflected grade-related compliance rather than self-motivated engagement. For these reasons, weekly check-in score was used as a supplementary behavioral indicator and examined in sensitivity analyses, rather than as the primary measure of GBE.

Table 2 provides a consolidated summary of the key procedural features of the Shanbay-based gamified vocabulary-learning task, including the platform, target vocabulary, weekly learning requirements, session-level settings, monitoring procedure, and check-in scoring arrangement.

**Table 2:** Summary of the Shanbay-based gamified vocabulary-learning procedure.

Aspect	Description
Setting	Required out-of-class vocabulary-learning task in the compulsory *College English I* course
Platform & target vocabulary	Shanbay app; CET-4 Syllabus Vocabulary List (abridged version), including 2,060 target words
Participants & duration	6 intact first-year non-English-major classes; 13 weeks
Task consistency	All students used the same app, word list, weekly learning load, and monitoring procedure
Weekly learning requirement	160 new words per week; minimum of four learning days per week
Session-level setting	40 newly assigned words and 80 review words per session, with a 1:2 newly assigned-to-review word ratio
Scheduling flexibility	Students could choose any 4 days within each week to complete the required sessions
Teacher instructions	Instructors explained the app-setting procedure, weekly requirement, weekly check-in documentation procedure, and assessment relevance
Monitoring procedure	Class monitors collected available weekly check-in evidence or completion information and recorded documented participation
Check-in scoring	Each valid daily check-in record was awarded 2 points; missing records received 0 points
Gamified affordances	Daily check-ins, task quantification, achievement- or badge-like rewards, progress tracking, and immediate feedback

“Newly assigned words” refers to words first presented to learners within the Shanbay learning plan. Some of these words may have already been known to students before the intervention.

At Time 2 (Week 13), participants completed the adapted GBE questionnaire and then took the vocabulary post-test during the same class session. The GBE instrument captured learners’ retrospective evaluations of their sustained effort, persistence, attention, and task involvement across the semester. A retrospective self-report design was considered appropriate because GBE in the present study refers to learners’ perceived behavioral investment across an extended learning period rather than momentary task engagement at a single point. The temporal separation of predictor measurement (IL2S at Time 1) from mediator and outcome measurement (GBE and vocabulary achievement at Time 2) also helped reduce common method concerns (Podsakoff et al., 2003) while allowing examination of a time-lagged statistical indirect association consistent with the proposed model.

### 3.4 Data analysis

Data analysis was conducted in three stages. Since no separate pilot testing was conducted before the main study, a *post-hoc* item analysis was performed using main-study data to evaluate the reliability, item difficulty, item discrimination, and empirical comparability of the researcher-developed vocabulary pre-test and post-test, thereby providing supplementary evidence regarding the quality of the two curriculum-based measures. Given that items were dichotomously scored as correct or incorrect, internal consistency reliability was estimated using the Kuder–Richardson Formula 20 (KR-20; Kuder and Richardson, 1937), which is mathematically equivalent to Cronbach’s alpha under binary scoring conditions (Cronbach, 1951). Both indices are reported for transparency.

Item difficulty was operationalized as the proportion of students answering each item correctly (the *p*-value), with higher values indicating easier items. Following Ebel and Frisbie (1991), items with *p* values below 0.30 were classified as difficult, items between 0.30 and 0.80 as moderate, and items above 0.80 as easy. Although the optimal difficulty range for maximizing item discrimination in norm-referenced contexts is approximately 0.40–0.60 (Lord, 1952), the present tests were curriculum-based mastery-oriented achievement measures rather than norm-referenced instruments, and higher mean difficulty values were therefore expected and appropriate given that student performance reflects the cumulative effect of repeated instruction and practice rather than a single-occasion ability estimate (Ebel and Frisbie, 1991).

Item discrimination was examined using corrected item-total correlations, with values at or above 0.20 interpreted as acceptable, values below 0.20 as weak, and negative values as warranting examination (Ebel and Frisbie, 1991). Two considerations are relevant to interpreting the discrimination indices obtained in the present study. First, sample homogeneity in intact classroom samples restricts total score variance and systematically attenuates corrected item-total correlations, without implying poor item construction (Crocker and Algina, 1986; Thorndike, 1949). Second, items approaching ceiling difficulty (*p* > 0.90) produce near-zero response variance, rendering their corrected item-total correlations statistically unstable and susceptible to negative values as a mathematical artifact rather than as evidence of substantive item malfunction (Crocker and Algina, 1986; Lord, 1952). In mastery-oriented classroom tests, a proportion of ceiling items is expected following instruction (Ebel and Frisbie, 1991). For each test form, KR-20, Cronbach’s *α*, mean item difficulty, difficulty range, mean corrected item-total correlation, discrimination range, and the number of negative-discrimination items were calculated to evaluate test reliability, item functioning, and the empirical comparability of the two forms.

Second, descriptive statistics and bivariate correlations among IL2S, self-reported GBE, vocabulary achievement, vocabulary pre-test scores, and aggregated weekly check-in score were examined. Because the weekly check-in records represented a coarse behavioral indicator of students’ routine app-based participation, their correlation with self-reported GBE was analyzed as supplementary behavioral evidence rather than as a direct substitute for the GBE scale. Since participants were nested within six intact classes, intraclass correlations were first calculated for the main study variables. Given the small number of clusters, class membership was then handled through a sensitivity analysis in which five class dummy variables were included as observed covariates predicting GBE and vocabulary post-test performance.

Sensitivity analyses were additionally conducted using aggregated weekly check-in score. In the first model, check-in score was added to the SEM model as an observed covariate to examine whether the main paths involving self-reported GBE remained after controlling for students’ recorded app-based participation. In the second model, check-in score replaced self-reported GBE as the intermediate behavioral indicator. Together, these analyses were used to assess whether the main findings were dependent on the self-reported engagement measure and to clarify whether the coarse behavioral record captured the same engagement-related process as the GBE scale.

**Figure 1:**
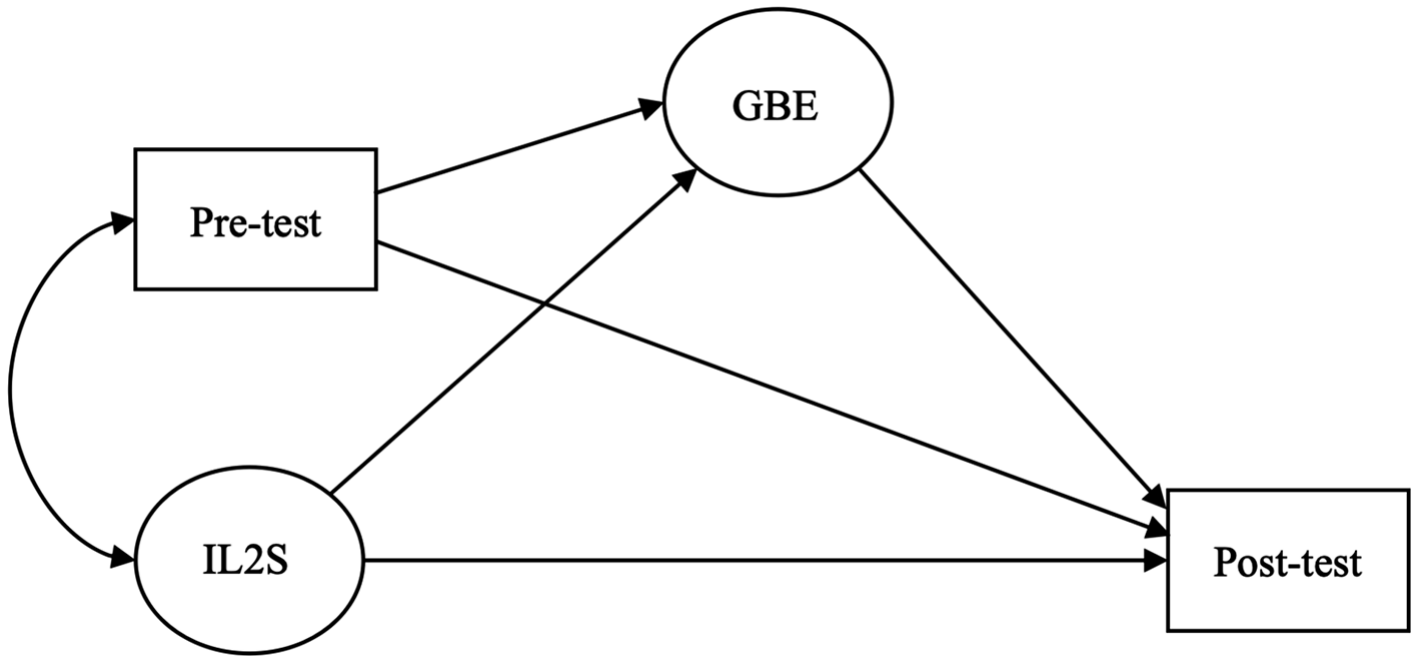
Conceptual indirect association model. IL2S stands for Ideal L2 Self; GBE represents Gamified Behavioral Engagement; Pre-test and Post-test represent participants’ scores on the vocabulary pre-test and post-test, respectively.

Third, structural equation modeling (SEM) was performed using AMOS 31 to test the hypothesized indirect-association model (Figure 1), including the association between IL2S and GBE, the association between GBE and vocabulary achievement, and the statistical indirect association between IL2S and vocabulary achievement through GBE. Vocabulary pre-test scores were specified as an observed covariate to control for baseline vocabulary knowledge when estimating the direct and indirect associations. Model fit was evaluated using multiple indices, including χ^2^/df, CFI, TLI, RMSEA, and SRMR, and the significance of indirect associations was examined via bootstrapping with bias-corrected confidence intervals. All statistical analyses were conducted using SPSS 31 and AMOS 31.

## 4 Results

### 4.1 Vocabulary test quality and comparability

A *post-hoc* item analysis was conducted to evaluate the reliability, item difficulty, item discrimination, and comparability of the researcher-developed vocabulary pre-test and post-test. As shown in Table 3, both tests contained 50 items with a possible score range of 0 to 100. The pre-test yielded a mean score of 59.92 (SD = 7.38) and the post-test a mean score of 72.92 (SD = 7.47). Internal consistency estimates were KR-20 = 0.628 and Cronbach’s *α* = 0.626 for the pre-test, and KR-20 = 0.697 and Cronbach’s α = 0.696 for the post-test. These values are consistent with levels typically observed in teacher-made, curriculum-based assessments administered to intact classroom samples (Quaigrain and Arhin, 2017).

**Table 3:** Reliability, item difficulty, and item discrimination indices for the pre- and post-test.

Index	Pre-test	Post-test
Number of items	50	50
Possible score range	0–100	0–100
Mean score (SD)	59.92 (7.38)	72.92 (7.47)
KR-20/Cronbach’s α	0.628/0.626	0.697/0.696
Mean item difficulty	0.599	0.729
Item difficulty range	0.110–1.000	0.050–1.000
Difficult items, *p* < 0.30	17	10
Moderate items, 0.30 ≤ *p* ≤ 0.80	11	11
Easy items, *p* > 0.80	22	29
Mean corrected item-total correlation	0.162	0.221
Discrimination range	−0.166–0.420	−0.075–0.542
Negative-discrimination items	11	5

Item difficulty was calculated as the proportion of correct responses; therefore, higher values indicate easier items. Item discrimination was indexed by corrected item-total correlations. KR-20, Kuder–Richardson Formula 20.

Regarding item difficulty, the mean index was 0.599 for the pre-test and 0.729 for the post-test. The pre-test included 17 difficult items, 11 moderate items, and 22 easy items; the post-test included 10 difficult items, 11 moderate items, and 29 easy items. The higher mean difficulty and larger number of easy items in the post-test likely reflect students’ vocabulary gains after a semester of practice, as curriculum-based achievement tests aim to capture instructional attainment rather than maintain fixed difficulty across time. At the same time, the pattern indicates that the post-test functioned as a somewhat easier form, and the two forms should therefore not be interpreted as statistically equivalent parallel tests. For this reason, pre-test scores were retained as a baseline covariate in the subsequent structural model to reduce potential bias associated with initial vocabulary knowledge and form-difficulty differences.

The mean corrected item-total correlation was 0.162 for the pre-test and 0.221 for the post-test, with 11 pre-test items and five post-test items showing negative correlations. In classical test theory, corrected item-total correlation is a common index of item discrimination, indicating whether students who perform well on the overall test also tend to answer a given item correctly (Nunnally and Bernstein, 1994). The modest correlations observed, particularly in the pre-test, should be interpreted cautiously because restricted score variance in intact classroom samples and extreme item difficulty can attenuate item-total correlations and render discrimination indices statistically unstable (Crocker and Algina, 1986; Ebel and Frisbie, 1991). Retaining items with weak or negative discrimination preserved the original scoring structure used in the classroom and avoided *post-hoc* alterations. Future studies should pilot larger item pools and revise or remove items with weak discrimination prior to formal administration.

Taken together, the reliability estimates, item difficulty distributions, and discrimination indices provide *post-hoc* evidence regarding the quality and comparability of the vocabulary measures. The two forms were comparable in structure, item format, scoring procedure, total score range, and CET-4-based content coverage. However, the post-test was somewhat easier and several items showed weak or negative discrimination; the two forms should therefore be regarded as comparable curriculum-based measures rather than strictly equivalent standardized parallel forms. For this reason, the pre-test score was retained as a covariate in the structural model.

### 4.2 Phase 2 measurement properties of IL2S and GBE

Before testing the structural model, the measurement properties of IL2S and GBE were examined using the Phase 2 field-study sample. This step was important because the structural model was estimated using the field-study data rather than the Phase 1 validation data. Therefore, the adequacy of the latent constructs needed to be evaluated within the same sample used for hypothesis testing. The two-factor measurement model showed acceptable fit to the data, χ^2^/df = 2.086, CFI = 0.955, TLI = 0.940, RMSEA = 0.078, and SRMR = 0.048. As shown in Table 4, standardized factor loadings ranged from 0.730 to 0.809 for IL2S and from 0.670 to 0.736 for GBE. Composite reliability (CR) was 0.892 for IL2S and 0.806 for GBE, and AVE values were 0.579 and 0.510, respectively. These results indicate acceptable internal consistency and convergent validity for both constructs in the main study.

**Table 4:** Measurement properties of IL2S and GBE in the main study.

Construct	Item	Standardized loading	CR	AVE
IL2S	IL2S1	0.730	0.892	0.579
	IL2S2	0.732		
	IL2S3	0.794		
	IL2S4	0.809		
	IL2S5	0.748		
	IL2S6	0.749		
GBE	GBE1	0.730	0.806	0.510
	GBE2	0.736		
	GBE3	0.719		
	GBE4	0.670		

IL2S, Ideal L2 Self; GBE, gamified behavioral engagement; CR, composite reliability; AVE, average variance extracted. Standardized factor loadings are reported based on the main study sample.

Discriminant validity was further examined using the Fornell–Larcker criterion. As shown in Table 5, the square roots of AVE were 0.761 for IL2S and 0.714 for GBE, both of which were higher than the latent correlation between the two constructs, *r* = 0.499. This pattern supports the discriminant validity of IL2S and GBE in the main study.

**Table 5:** Discriminant validity of IL2S and GBE.

Construct	IL2S	GBE
IL2S	0.761	
GBE	0.499	0.714

IL2S, Ideal L2 Self; GBE, gamified behavioral engagement. Diagonal values represent the square roots of AVE. The off-diagonal value represents the latent correlation between IL2S and GBE.

### 4.3 Descriptive statistics and correlation analysis between variables

Table 6 summarizes the descriptive statistics and zero-order correlations among the study variables. Participants reported relatively high levels of IL2S (*M* = 4.81, *SD* = 0.80) and GBE (*M* = 4.90, *SD* = 0.93), both exceeding the midpoint of their respective scales. Vocabulary post-test scores showed adequate dispersion (M = 72.92, SD = 7.47), indicating sufficient inter-individual variability for subsequent model estimation. However, as reported in the preceding item analysis, the post-test was somewhat easier than the pre-test. Therefore, subsequent SEM analyses controlled for vocabulary pre-test scores to account for baseline vocabulary knowledge and to reduce potential bias associated with form-difficulty differences.

**Table 6:** Descriptive statistics and correlations among study variables (*N* = 181).

Variables	Mean	SD	Skewness	Kurtosis	IL2S	GBE	Pre-test	Post-test	Check-in
IL2S	4.81	0.80	−0.49	−0.57	-				
GBE	4.90	0.93	−0.37	0.19	0.42**	-			
Pre-test	59.92	7.38	0.31	−0.23	0.39**	0.44**	-		
Post-test	72.92	7.47	0.14	−0.35	0.43**	0.49**	0.91**	-	
Check-in	62.00	9.12	−0.41	6.67	0.11	0.25**	0.135	0.162*	-

Check-in score was based on documented daily check-ins, with each valid check-in awarded 2 points. It was treated as a coarse supplementary behavioral indicator rather than a complete objective app-use log. *p < 0.05, **p < 0.01.

Correlation analyses indicated that IL2S was positively associated with GBE (*r* = 0.42, *p* < 0.01) and vocabulary post-test achievement (*r* = 0.43, *p* < 0.01). GBE was also positively correlated with vocabulary achievement (*r* = 0.49, *p* < 0.01). Vocabulary pre-test scores demonstrated a strong correlation with post-test performance (*r* = 0.91, *p* < 0.01), reflecting substantial rank-order stability in learners’ vocabulary knowledge across the instructional period. Such strong associations are consistent with the cumulative nature of vocabulary acquisition, in which prior lexical knowledge often serves as a powerful predictor of subsequent vocabulary performance (Lin and Lin, 2019; Nation, 2022).

In addition, vocabulary pre-test scores were moderately correlated with both IL2S (*r* = 0.39, *p* < 0.01) and GBE (*r* = 0.44, *p* < 0.01), suggesting shared variance between baseline proficiency, motivational self-guides, and behavioral engagement. Overall, the pattern of correlations was consistent with the hypothesized relationships and supported the subsequent SEM analyses.

As a supplementary analysis, aggregated weekly check-in score was examined as a coarse behavioral indicator of students’ routine app-based participation during the intervention period (*M* = 62.00, *SD* = 9.12, Range = 10–90). It was positively correlated with self-reported GBE at a small but statistically significant level (*r* = 0.25, *p* < 0.01), suggesting that the check-in records captured a related but only partially overlapping aspect of behavioral engagement, which therefore were not included in the main structural model.

### 4.4 Class-level clustering and class-adjusted sensitivity analysis

Since participants were recruited from six intact teaching-administrative classes, intraclass correlations were calculated to examine the extent to which the main variables varied at the class level. As presented in Table 7, the ICC values ranged from 0.062 to 0.078 across IL2S, GBE, vocabulary pre-test, vocabulary post-test, and check-in score. These values indicate that a modest proportion of variance was attributable to class membership. Although the ICC values suggested modest class-level clustering, they did not indicate severe dependence among observations. Because the data included only six intact classes, a full multilevel SEM was not estimated, as the number of clusters was insufficient for stable multilevel parameter estimation. Instead, a class-adjusted sensitivity model was conducted by including class membership dummy variables as observed covariates predicting GBE and vocabulary post-test performance. This approach was used to examine whether the main substantive paths remained robust after adjusting for class membership.

**Table 7:** Intraclass correlations for the main study variables.

Variable	ICC
IL2S composite score	0.078
GBE composite score	0.078
Pre-test	0.064
Post-test	0.071
Check-in	0.062

ICC, intraclass correlation coefficient. ICCs were calculated to examine the extent to which variance in the main study variables was attributable to class-level clustering. The participants were nested within six intact classes.

To further examine whether class-level clustering affected the substantive findings, a class-adjusted sensitivity model was estimated. Class membership was represented by five dummy variables, with Class 1 as the reference group. These class dummy variables were included as observed covariates predicting both GBE and vocabulary post-test performance. The class-adjusted model showed acceptable fit, χ^2^/df = 1.845, CFI = 0.945, TLI = 0.917, RMSEA = 0.069, SRMR = 0.049. As showcased in Table 8, the main pattern of results remained unchanged. IL2S significantly predicted GBE, *β* = 0.318, *p* = 0.005, and GBE significantly predicted vocabulary post-test performance, *β* = 0.128, *p* = 0.007. The direct path from IL2S to post-test performance remained non-significant, *β* = 0.053, *p* = 0.193. The indirect effect of IL2S on vocabulary post-test performance through GBE also remained statistically significant, *β* = 0.041, 95% BC CI [0.009, 0.096], *p* = 0.007. These results suggest that the main indirect-effect pattern was not eliminated after adjusting for class membership.

**Table 8:** Class-adjusted sensitivity analysis controlling for class membership.

Path	Standardized *β*	*p*	95% BC CI	Result
IL2S → GBE	0.318	0.005	[0.102, 0.521]	Significant
GBE → Post-test	0.128	0.007	[0.037, 0.208]	Significant
IL2S → Post-test	0.053	0.193	[−0.030, 0.132]	Not Significant
Pre-test → Post-test	0.821	< 0.001	[0.753, 0.876]	Significant
IL2S → GBE → Post-test	0.041	0.007	[0.009, 0.096]	Significant

IL2S, Ideal L2 Self; GBE, gamified behavioral engagement; BC CI, bias-corrected confidence interval. Class membership was represented by five dummy variables, with Class 1 used as the reference group. The class dummy variables were included as observed covariates predicting GBE and vocabulary post-test performance. Standardized coefficients are reported. The standardized indirect effect was estimated using 5,000 bootstrap samples.

### 4.5 Sensitivity analyses using weekly check-in score

To further examine whether the main findings were dependent on the use of self-reported GBE, two sensitivity analyses were conducted using aggregated weekly check-in score as a supplementary behavioral indicator. Weekly check-in score was treated as a coarse record of routine app-based participation rather than as an equivalent substitute for self-reported GBE, because it indicated whether students completed the required weekly participation but did not capture the quality, intensity, or psychological meaning of their engagement.

As shown in Table 9, in Model A, weekly check-in score was added to the original SEM model as an observed covariate predicting both self-reported GBE and vocabulary post-test performance. The model showed acceptable fit to the data, χ^2^/df = 1.833, CFI = 0.959, TLI = 0.946, SRMR = 0.057, RMSEA = 0.068. After check-in score was included, IL2S continued to significantly predict self-reported GBE, *β* = 0.349, *p* = 0.001, 95% BC CI [0.135, 0.545], and self-reported GBE remained a significant positive predictor of vocabulary post-test performance, *β* = 0.115, *p* = 0.017, 95% BC CI [0.028, 0.197]. The indirect effect of IL2S on vocabulary post-test performance through self-reported GBE also remained statistically significant, *β* = 0.040, 95% BC CI [0.010, 0.096], *p* = 0.007. Weekly check-in score significantly predicted self-reported GBE, *β* = 0.203, *p* = 0.045, 95% BC CI [0.004, 0.419], but did not significantly predict vocabulary post-test performance after controlling for pre-test performance, IL2S, and self-reported GBE, *β* = 0.012, *p* = 0.665, 95% BC CI [−0.043, 0.069].

**Table 9:** Sensitivity analyses involving weekly check-in score.

Model	Path	β	*p*	95% BC CI	Result
Model A: Check-in as an observed covariate	IL2S → GBE	0.349	0.001	[0.135, 0.545]	Significant
	GBE → Post-test	0.115	0.017	[0.028, 0.197]	Significant
	Check-in → GBE	0.203	0.045	[0.004, 0.419]	Significant
	Check-in → Post-test	0.012	0.665	[−0.043, 0.069]	Not significant
	IL2S → GBE → Post-test	0.040	0.007	[0.010, 0.096]	Significant
Model B: Check-in replacing GBE as mediator	IL2S → Check-in	0.068	0.349	[−0.082, 0.218]	Not significant
	Check-in → Post-test	0.035	0.219	[−0.019, 0.086]	Not significant
	IL2S → Check-in → Post-test	0.002	0.253	[−0.002, 0.015]	Not significant

IL2S, Ideal L2 Self; GBE, gamified behavioral engagement; BC CI, bias-corrected confidence interval. Standardized coefficients are reported. Model A added weekly check-in score as an observed covariate predicting both self-reported GBE and vocabulary post-test performance. Model B replaced self-reported GBE with weekly check-in score as the mediator between IL2S and vocabulary post-test performance. Pre-test performance was included as a covariate in both models.

With regard to Model B, weekly check-in score replaced self-reported GBE as an intermediate indicator of the indirect association between IL2S and vocabulary post-test performance, with pre-test performance included as a covariate. This alternative model showed good fit to the data, χ^2^/df = 1.718, CFI = 0.981, TLI = 0.971, RMSEA = 0.063. The results did not provide statistical evidence for the hypothesized mediation pattern when check-in score was used as the mediator. IL2S did not significantly predict weekly check-in score, *β* = 0.068, *p* = 0.349, 95% BC CI [−0.082, 0.218], and weekly check-in score did not significantly predict vocabulary post-test performance, *β* = 0.035, *p* = 0.219, 95% BC CI [−0.019, 0.086]. The indirect effect of IL2S on vocabulary post-test performance through check-in score was also non-significant, *β* = 0.002, 95% BC CI [−0.002, 0.015], *p* = 0.253. In contrast, pre-test performance remained a significant predictor of vocabulary post-test performance, *β* = 0.866, *p* = 0.001, 95% BC CI [0.813, 0.900].

Overall, Model A showed that the key GBE-related paths remained significant after weekly check-in score was included as an observed covariate, whereas Model B showed that weekly check-in score did not serve as a significant alternative mediator.

### 4.6 Structural equation modeling results

SEM was conducted to examine the hypothesized relationships among IL2S, GBE, and vocabulary achievement, while controlling for baseline vocabulary knowledge. The proposed indirect-association model demonstrated an acceptable fit to the data, with χ^2^/df = 1.789, CFI = 0.967, TLI = 0.956, RMSEA = 0.066, and SRMR = 0.046, indicating that the specified model adequately represented the observed covariance structure.

Table 10 presents the unstandardized and standardized path estimates of the structural model. IL2S showed a significant positive association with GBE (*β* = 0.359, *p* < 0.001), indicating that learners with a stronger IL2S tended to report higher levels of sustained behavioral engagement in gamified vocabulary learning activities. Vocabulary pre-test scores demonstrated a very strong correlation with post-test performance (*r* = 0.91, *p* < 0.01), indicating substantial rank-order stability in learners’ vocabulary performance across the semester. This pattern suggests that a large proportion of post-test variance reflected prior vocabulary knowledge. Therefore, the SEM results involving GBE should be interpreted as baseline-adjusted associations, and the contribution of GBE should be understood as incremental to, rather than independent of, students’ initial vocabulary knowledge.

**Table 10:** Standardized and unstandardized path estimates of the structural model.

Structural path	Estimate	SE	*β*	*p*
IL2S → GBE	0.437	0.110	0.359	< 0.001
Pre-test → GBE	0.038	0.009	0.340	< 0.001
GBE → Post-test	1.097	0.407	0.120	0.007
IL2S → Post-test	0.577	0.436	0.052	0.185
Pre-test → Post-test	0.839	0.036	0.830	< 0.001

Estimates are unstandardized coefficients. β denotes standardized path coefficients. All paths were estimated while controlling for baseline vocabulary knowledge.

GBE, in turn, showed a small but statistically significant positive association with vocabulary post-test achievement (*β* = 0.120, *p* = 0.007). By contrast, the direct path from IL2S to vocabulary post-test achievement was not statistically significant (*β* = 0.052, *p* = 0.185) once behavioral engagement and baseline vocabulary knowledge were taken into account. As expected, vocabulary pre-test scores showed a strong positive association with post-test performance (*β* = 0.830, *p* < 0.001), underscoring the substantial contribution of prior vocabulary knowledge to subsequent achievement.

**Figure 2:**
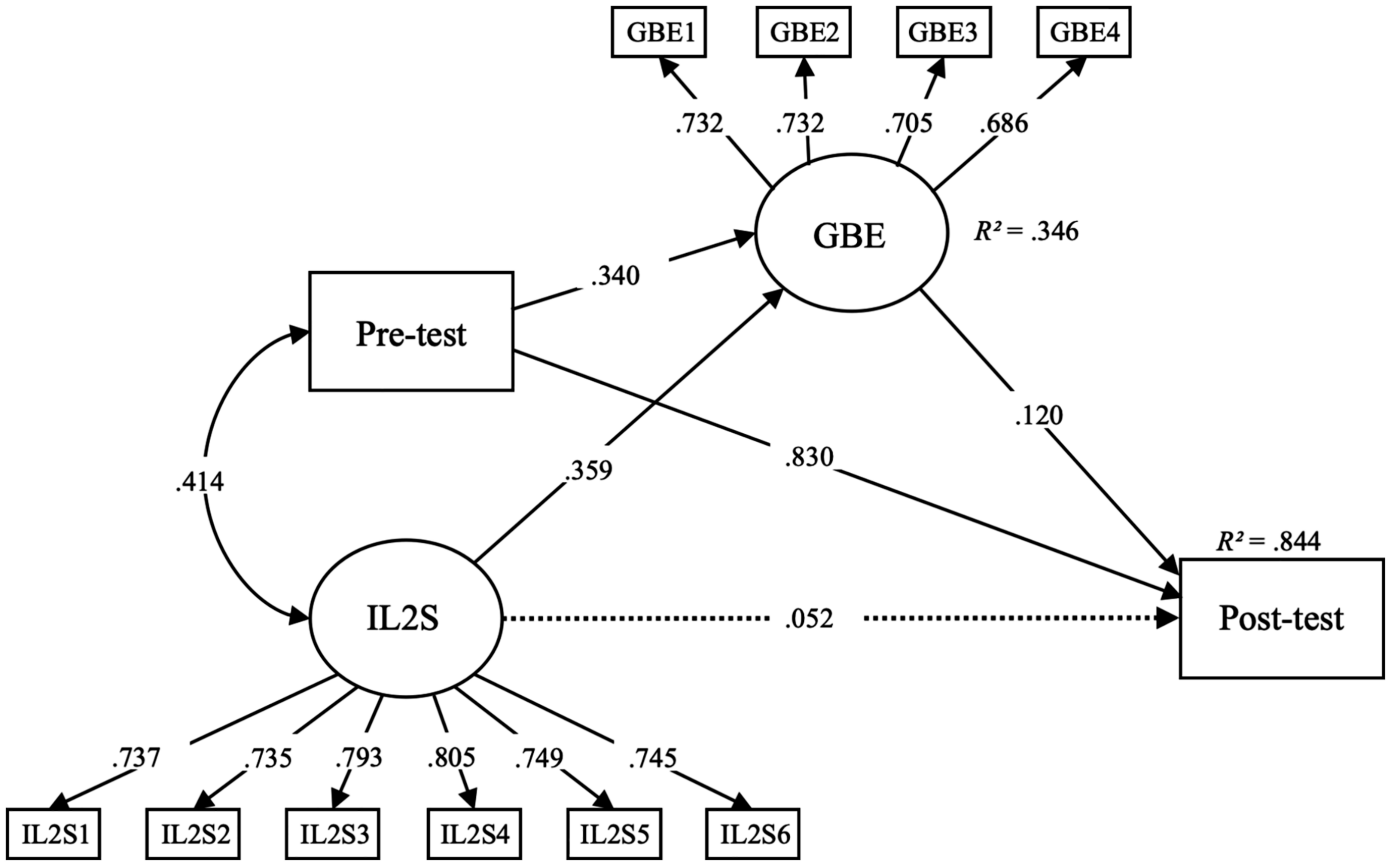
The standardized path coefficient results of the model illustrating indirect associations. IL2S = Ideal L2 Self; GBE = gamified behavioral engagement. Standardized coefficients are shown. Ellipses indicate latent variables, and rectangles indicate observed variables. Solid lines represent significant paths, and the dotted line represents a non-significant path. Residual terms are omitted for clarity.

The explanatory power of the model is summarized in Table 11. The model accounted for approximately 34.6% of the variance in GBE and 84.4% of the variance in vocabulary post-test achievement. To account for shared variance among exogenous variables, a covariance was specified between IL2S and vocabulary pre-test scores, reflecting their empirical association and improving overall model representation without implying a causal relationship. Figure 2 illustrates the final structural model with standardized path coefficients.

**Table 11:** Explained variance (squared multiple correlations) of endogenous variables.

Endogenous variable	*R^2^*
Gamified behavioral engagement	0.346
Post-test achievement	0.844

R^2^ values indicate the proportion of variance explained by the predictors specified in the model.

### 4.7 Indirect association via GBE

To examine whether IL2S was statistically and indirectly associated with vocabulary achievement through GBE, indirect associations were tested using bias-corrected bootstrapping with 5,000 resamples within the SEM framework, while controlling for baseline vocabulary knowledge. The results of the indirect-effect analysis are summarized in Table 12.

**Table 12:** Direct, indirect, and total associations of ideal L2 self and vocabulary achievement.

Effect type	Estimate	β	*p*	95% BC CI
Direct effect (IL2S → Post-test)	0.577	0.052	*p* = 0.185	[−0.023, 0.125]
Indirect effect (IL2S → GBE → Post-test)	0.479	0.043	*p* = 0.003	[0.012, 0.096]
Total effect	1.056	0.095	*p* = 0.005	[0.032, 0.164]

Indirect effects were tested using bias-corrected bootstrapping with 5,000 resamples. CI, confidence interval.

The statistical indirect association between IL2S and vocabulary post-test achievement through GBE was significant. Although the standardized indirect association was modest in magnitude, it remained statistically reliable after controlling for baseline vocabulary knowledge. Specifically, the unstandardized indirect estimate was 0.479, with a standardized effect of *β* = 0.043, and the 95% bias-corrected confidence interval did not include zero ([0.012, 0.096]), providing evidence for a statistically significant indirect association through behavioral engagement. In contrast, the direct effect of IL2S on vocabulary post-test achievement remained non-significant after accounting for GBE and prior vocabulary knowledge (*β* = 0.052, *p* = 0.185).

Taken together, these findings are consistent with a statistically significant indirect-association pattern. However, given the modest size of the indirect effect and the strong contribution of baseline vocabulary knowledge, the finding should be interpreted as indicating that self-reported GBE added a small but statistically reliable contribution to vocabulary achievement, rather than serving as the primary explanation of post-test performance.

## 5 Discussion

### 5.1 IL2S as a motivational antecedent of GBE

The present study found that IL2S was positively associated with GBE in the gamified MAVL context. This result is consistent with the L2MSS, in which IL2S is conceptualized as a future-oriented motivational resource that helps direct learners’ sustained effort toward language learning goals (Dörnyei, 2005; Boo et al., 2015). In the current study, learners who reported a stronger idealized image of themselves as future English users also reported higher levels of persistence, effort, and continued involvement in app-based vocabulary learning activities.

This finding also echoes earlier discussion in the literature review that motivational self-guides are more likely to be associated with learning outcomes through more proximal forms of learner involvement rather than through a direct route alone. In particular, Oga-Baldwin et al. (2019) describe engagement as a central construct linking learners’ prior beliefs and experiences with later outcomes, while Gong and Pang (2025) likewise showed that the ideal L2 writing self was positively related to multiple dimensions of engagement, including behavioral engagement. Although those studies were not conducted in a mobile vocabulary-learning context, the present result is broadly consistent with their shared implication that a desired future L2 self may matter partly because it is associated with learners’ active participation in learning tasks.

At the same time, the present finding should be interpreted with appropriate caution. The result does not suggest that IL2S directly guarantees vocabulary success, nor does it imply that all gamified learning environments necessarily strengthen this motivational process in the same way. As discussed in the literature review, gamified environments vary considerably across contexts and design features, and their effects are not uniformly positive or equally strong across outcomes (Alsawaier, 2018; Koivisto and Hamari, 2019; Sailer and Homner, 2020). Accordingly, the present study supports the more limited claim that, in this specific MAVL setting, IL2S functioned as a motivational antecedent that was positively related to self-reported behavioral engagement.

### 5.2 GBE and vocabulary achievement

The present study also found that GBE was positively associated with vocabulary post-test performance, although the standardized effect was relatively small. Importantly, the statistical significance of this path should be distinguished from its practical or educational significance. The small standardized coefficient indicates that GBE did not account for a large proportion of vocabulary post-test performance, especially when compared with the much stronger role of vocabulary pre-test scores. Educationally, this result suggests that self-reported behavioral engagement provided a meaningful but limited contribution to vocabulary achievement by supporting sustained participation in repeated vocabulary practice. It should therefore not be interpreted as evidence that engagement was the dominant determinant of vocabulary achievement. This result is in line with the view that behavioral engagement reflects a relatively proximal form of learner involvement, especially in learning contexts that depend on repeated practice over time (Fredricks et al., 2004; Philp and Duchesne, 2016). In MAVL, such repeated involvement is particularly relevant because vocabulary development relies on repeated exposure, retrieval, and cumulative practice rather than on one-time task completion (Nation, 2022; Lin and Lin, 2019; Hao et al., 2021; Waluyo and Bucol, 2021). This interpretation is also broadly compatible with prior research on gamified English vocabulary learning showing that gamified conditions can be associated with stronger vocabulary outcomes as well as more positive motivational and satisfaction-related responses, although such findings do not by themselves establish behavioral engagement as the operative mechanism (Yu, 2023).

This finding also broadly echoes the cautious position developed in the literature review. Previous research has suggested that sustained involvement in app-based vocabulary learning can support vocabulary development, but the strength of the engagement–achievement relationship is not always uniform across studies or outcome measures (He and Loewen, 2022; Zhou and Zhou, 2026). In the present study, the positive path from GBE to vocabulary achievement is therefore best interpreted as evidence that learners’ reported persistence and continued participation were meaningfully associated with learning outcomes, rather than as evidence that engagement alone strongly determines achievement. It should also be noted that this association was observed in a specific instructional context — a convenience sample of first-year non-English-major students at a single private university — and cannot be assumed to generalize across different learner populations, institutional settings, or application types. The gender imbalance in the sample (73.48% female) further limits the generalizability of this finding, as motivational self-guides and behavioral engagement may function differently across gender groups.

The relatively modest size of this association should also be understood in light of another clear result from the structural model: vocabulary pre-test scores showed a very strong positive relationship with post-test performance. This pattern is consistent with the cumulative nature of vocabulary learning and suggests that prior lexical knowledge remained a major source of variance in later achievement (Nation, 2022; Lin and Lin, 2019; Hao et al., 2021). Against this background, the present result suggests that self-reported behavioral engagement made an additional, statistically significant contribution to vocabulary achievement, but one that was smaller than the contribution of baseline vocabulary knowledge.

This interpretation should also be considered alongside the measurement characteristics of the vocabulary tests. As reported in the *post-hoc* item analysis, the post-test was somewhat easier than the pre-test, and the two forms cannot be treated as strictly equivalent standardized parallel tests. Therefore, the positive association between GBE and post-test performance should be interpreted as a baseline-adjusted association based on comparable curriculum-based measures rather than as evidence derived from fully equivalent standardized test forms.

The sensitivity analyses further clarify the role of self-reported GBE in the model. When weekly check-in score was added as an observed covariate, the path from self-reported GBE to vocabulary achievement and the indirect effect through GBE remained significant. However, when check-in score replaced self-reported GBE as the mediator, neither the motivational path from IL2S to check-in score nor the achievement path from check-in score to post-test performance was significant. This pattern suggests that the main findings were not simply a function of recorded app participation. Rather, self-reported GBE appeared to capture a broader form of engagement involving learners’ perceived effort, attention, persistence, and sustained task involvement. By contrast, check-in score may have reflected a more compliance-oriented indicator of participation, especially because weekly check-ins counted toward the course grade. Therefore, the results support the interpretation that self-reported GBE and check-in score are complementary but non-interchangeable indicators of learner involvement in gamified vocabulary learning.

### 5.3 GBE as an indirect pathway linking IL2S and vocabulary achievement

A central finding of the present study is that IL2S showed a statistically significant indirect association with vocabulary achievement through self-reported GBE, whereas the direct path from IL2S to vocabulary achievement was not statistically significant after GBE and baseline vocabulary knowledge were added into the model. This pattern is best interpreted as an indirect statistical association rather than as evidence of a causal mediation process. More specifically, the findings suggest that learners with a stronger future-oriented motivational self-guide tended to report higher behavioral involvement in gamified vocabulary learning activities, and this reported involvement was, in turn, associated with higher vocabulary post-test performance.

This interpretation is consistent with the theoretical position outlined in the literature review. Within the L2MSS framework, IL2S is a relatively distal motivational construct, whereas engagement represents a more immediate form of learner involvement in actual learning activity (Dörnyei, 2005; Boo et al., 2015; Oga-Baldwin et al., 2019). More broadly, recent EFL evidence suggests that engagement may function as a proximal correlate of achievement through which contextual resources are associated with achievement (Tang et al., 2026), further situating engagement as a near-term process variable rather than a distal one. Likewise, prior studies reviewed earlier suggest that future self-guides are more meaningfully connected to performance when they are considered alongside learners’ behavioral participation in learning tasks (Gong and Pang, 2025; Zhan et al., 2023). The present results are therefore broadly compatible with the idea that motivation may be associated with achievement partly through engagement-related indicators.

At the same time, this indirect pathway should not be interpreted too strongly. Since GBE and vocabulary post-test scores were assessed at the same time point, the present results are better understood as supporting a statistically significant indirect association rather than establishing a definitive causal mediating mechanism. In addition, the size of the standardized indirect effect was modest. For this reason, the present study does not show that IL2S automatically leads to higher achievement, nor does it demonstrate that gamification itself directly produces vocabulary gains through a single behavioral mechanism. Rather, the findings support the more cautious conclusion that, in this MAVL context, learners with a stronger IL2S tended to report greater behavioral engagement, and this higher engagement was associated with better vocabulary performance.

Finally, this result also speaks to the broader concern raised in the literature review that research on gamified learning has often identified positive motivational or learning outcomes without clearly specifying the processes connecting them (Alsawaier, 2018; Koivisto and Hamari, 2019). The present study does not fully resolve that issue, but it does provide evidence that self-reported behavioral engagement is one plausible engagement-related link in the statistical association between a motivational resource such as IL2S and vocabulary achievement in a gamified mobile-assisted learning environment.

## 6 Pedagogical implications

The present findings offer several implications for vocabulary teaching in gamified mobile-assisted learning contexts. First, the positive association between IL2S and GBE suggests that vocabulary instruction may benefit from not only providing learners with repeated practice opportunities, but also helping them connect such practice to their personally meaningful future L2 goals. In practice, teachers may encourage learners to reflect on what kind of English user they hope to become and to consider how sustained vocabulary practice relates to their longer-term academic or professional aspirations, thereby making repeated engagement with gamified tasks feel personally relevant rather than merely externally required. For instance, short reflective prompts embedded at the beginning or end of a learning session—asking learners to articulate how their current vocabulary practice connects to their future goals as L2 users—may help reinforce this sense of personal relevance across the semester. This implication should be understood cautiously: the present study does not suggest that future self-guides alone are sufficient to improve achievement, but it does indicate that they may help support learners’ sustained involvement in vocabulary learning activities.

Second, the significant but modest association between GBE and vocabulary achievement suggests that teachers should pay attention to sustaining regular behavioral participation in vocabulary learning, especially in contexts where lexical development depends on cumulative practice over time. In this respect, gamified mobile-assisted vocabulary learning may be pedagogically useful when it helps students maintain consistent practice routines, such as repeated review, continued task completion, and persistent engagement across the semester. Relatedly, Shen et al. (2024) indicate that technology-enhanced EFL tasks may support engagement when they provide interactive and multimodal learning experiences, implying that points, progress indicators, and feedback should be embedded in tasks that require active attention and repeated participation, rather than being used as superficial motivational add-ons. However, the relatively small size of the effect also indicates that engagement should not be treated as the sole determinant of learning outcomes. As the strong role of vocabulary pre-test scores in the present study shows, prior knowledge remains a major factor in later performance. Accordingly, gamified engagement should be viewed as a supportive component of vocabulary instruction rather than a substitute for systematic vocabulary teaching, appropriate task design, or learners’ existing lexical foundation. More broadly, recent EFL evidence suggests that engagement can be pedagogically supported rather than left entirely to learner initiative (Tang et al., 2026). In practice, this may mean that teachers periodically monitor students’ app-based participation, provide encouragement or task-level guidance to students whose participation appears to decline, and integrate gamified vocabulary routines with in-class review activities so that app-based practice is reinforced rather than treated as an isolated self-study component (Waluyo and Bucol, 2021). Therefore, the practical value of gamified behavioral engagement lies less in producing large achievement gains by itself and more in helping learners maintain regular, effortful, and sustained vocabulary practice over time.

Third, the indirect association found in this study suggests that teachers may gain more from considering how motivational self-guides are accompanied by day-to-day learning behavior than from assuming that motivational intensity alone will directly lead to vocabulary gains. From a pedagogical perspective, this means that instructional attention should be directed not only to whether students value English learning, but also to whether classroom and app-based practices encourage them to persist in actual learning activities. In other words, pedagogical support may be more effective when motivational resources and behavioral participation are considered together.

At the same time, these implications should be interpreted within the boundaries of the present study. Because the indirect pathway identified here is statistical rather than definitively causal, the findings do not justify strong claims that gamification itself directly produces vocabulary improvement through a single mechanism. Nor do they suggest that all gamified applications will work equally well across learners or contexts. Instead, the more defensible implication is that, in this particular MAVL setting, gamified vocabulary learning appears to be more pedagogically meaningful when it is accompanied by learners’ future-oriented motivation and sustained behavioral participation.

Overall, the present study suggests that effective pedagogical use of gamified mobile-assisted vocabulary learning should combine three considerations: the cultivation of learners’ future-oriented L2 goals, the support of regular and sustained behavioral engagement, and continued attention to learners’ existing vocabulary knowledge. A balanced instructional approach of this kind is likely to be more appropriate than relying on gamified features alone to improve vocabulary achievement.

These findings, however, need to be interpreted within the contextual boundaries of the study design. Phase 2 was a classroom-based observational study using a convenience sample from six intact classes at a single private university in eastern China. The sample was also gender-imbalanced, with female students substantially outnumbering male students. Accordingly, the results should be understood as context-specific, baseline-adjusted associations rather than as causal or broadly generalizable effects of gamified mobile-assisted vocabulary learning. These limitations do not invalidate the observed pattern, but they narrow the strength and generalizability of the conclusions.

## 7 Limitations and future research

Several limitations of the present study should be acknowledged. First, although the study adopted a two-wave design in which IL2S was measured at the beginning of the semester and both GBE and vocabulary post-test performance were assessed at the end of the intervention, GBE and achievement were measured at the same time point. As a result, the identified indirect pathway should be interpreted as a statistically significant indirect association rather than definitive evidence of a causal mediating mechanism. Future research could strengthen temporal inference by employing three-wave or longitudinal designs in which motivational variables, engagement, and achievement are measured at separate time points.

Second, behavioral engagement in the present study was primarily assessed through self-report. Although the supplementary weekly check-in records showed a small but significant positive correlation with self-reported GBE, these records captured only a coarse form of participation and did not reflect more fine-grained aspects of learner behavior, such as time on task, number of completed words, review frequency, item-level accuracy, response latency, or detailed review trajectories. In addition, weekly check-in completion accounted for 15% of the course grade, which may have encouraged some students to complete check-ins for grade-related compliance rather than as a direct expression of motivationally driven engagement. The sensitivity analyses partly addressed this concern by showing that the main indirect pathway through self-reported GBE remained significant after check-in score was controlled, whereas check-in score itself did not function as a significant intermediate indicator of the indirect association. Nevertheless, future studies should combine self-report measures with more detailed platform-generated learning analytics and, where possible, separate research measures of engagement from course assessment requirements.

Third, vocabulary achievement was measured using researcher-developed CET-4-based tests rather than a standardized external vocabulary assessment. Although the two forms followed the same test specifications, used the same item formats and scoring procedures, were reviewed by two experienced College English instructors, and were aligned with the same CET-4 vocabulary source, no separate pilot testing was conducted before the main study. The *post-hoc* item analysis provided useful information about reliability, item difficulty, item discrimination, and empirical comparability; however, it also indicated that the post-test was somewhat easier than the pre-test and that several items showed weak or negative discrimination. Therefore, the two tests should be regarded as comparable curriculum-based measures rather than strictly equivalent standardized parallel forms. To reduce the potential influence of baseline vocabulary knowledge and form-difficulty differences, pre-test vocabulary achievement was included as a covariate in the structural model. Nevertheless, future studies should further strengthen outcome measurement by piloting parallel test forms before the intervention, using larger item pools, matching target words more systematically, revising or removing items with unstable discrimination, and incorporating standardized or externally validated vocabulary measures where feasible.

Fourth, the study was conducted in a specific instructional setting involving first-year non-English-major students at a private university in eastern China and a single mobile-assisted vocabulary learning context. The Phase 2 sample was also gender-imbalanced, with female students accounting for a substantially larger proportion of participants than male students. This context-specific design supports ecological relevance, but it also limits the generalizability of the findings. Future research could examine whether the observed relationships hold across different institutional contexts, learner populations, gender-balanced samples, proficiency levels, and gamified applications.

Fifth, the indirect effect identified in this study was statistically significant but modest in magnitude, while baseline vocabulary knowledge remained a particularly strong predictor of post-test achievement. This suggests that the relationship among IL2S, GBE, and vocabulary achievement is only one part of a broader explanatory picture. Future research may therefore benefit from incorporating additional variables that were not modeled here, such as other dimensions of engagement, affective responses to gamified learning, learner autonomy, or instructional differences across teachers and classes. In the present study, all six classes used the same vocabulary list and app-based learning requirements; however, subtle differences in how individual instructors introduced, monitored, or discussed the learning task were not systematically documented and could not be ruled out as potential sources of variation in learners’ motivational and engagement responses. Incorporating such contextual factors in future designs would provide a more complete account of how motivational self-guides, behavioral engagement, and vocabulary achievement relate to one another in naturalistic instructional settings.

Finally, although the present study focused specifically on IL2S as a motivational antecedent, the broader motivational structure of learners’ self-guides was not examined in full. Given that previous research has distinguished among multiple self-related motivational dimensions, future studies may further clarify whether the role observed here is specific to IL2S or varies when other motivational self-guides are considered alongside behavioral engagement in gamified vocabulary learning contexts.

Overall, the present study provides evidence for this observed indirect association between IL2S, GBE, and vocabulary achievement in a gamified mobile-assisted vocabulary learning context, but the limitations noted above suggest that these findings should be interpreted with appropriate caution and extended through further research.

## 8 Conclusion

The present study examined the relationships among IL2S, GBE, and vocabulary achievement in a gamified mobile-assisted vocabulary learning context while controlling for baseline vocabulary knowledge. The findings showed that IL2S was positively associated with GBE, and that GBE, in turn, was positively associated with vocabulary post-test performance, although the effect size was modest. In addition, IL2S showed a statistically significant indirect association with vocabulary achievement through GBE, whereas the direct path from IL2S to vocabulary achievement was not significant once GBE and prior vocabulary knowledge were taken into account.

These findings suggest that the role of IL2S in vocabulary learning may be better understood in relation to learners’ sustained behavioral participation rather than as a direct predictor of achievement alone. At the same time, the results also indicate that prior vocabulary knowledge remained a particularly strong predictor of later performance, underscoring the cumulative nature of vocabulary development. Accordingly, the present study supports a cautious interpretation: in this specific MAVL context, learners’ future-oriented motivation was indirectly associated with vocabulary achievement via self-reported behavioral engagement, although this association was modest and should not be interpreted as evidence of a definitive causal mediation process. Furthermore, because GBE was assessed through self-report at a single retrospective time point, this measurement approach may have introduced recall bias or social desirability effects, as learners may have evaluated their engagement in ways influenced by their final course performance or overall satisfaction with the learning context. Relatedly, the absence of fine-grained platform-generated behavioral records means that the present findings cannot distinguish between engagement that was genuinely self-directed and participation that was partly compliance-driven due to the check-in grading requirement. These observational and measurement constraints narrow the strength of the inferences that can be drawn and underscore the need for designs that separate motivational, behavioral, and achievement measurement both temporally and methodologically.

## Data Availability

The original contributions presented in the study are included in the article/supplementary material, further inquiries can be directed to the corresponding author.
